# Optimization, characterization, comparison of self-assembly VLP of capsid protein L1 in yeast and reverse vaccinology design against human papillomavirus type 52

**DOI:** 10.1186/s43141-023-00514-9

**Published:** 2023-05-24

**Authors:** Moh Egy Rahman Firdaus, Apon Zaenal Mustopa, Nurlaili Ekawati, Sheila Chairunnisa, Rosyida Khusniatul Arifah, Ai Hertati, Shasmita Irawan, Anika Prastyowati, Arizah Kusumawati, Maritsa Nurfatwa

**Affiliations:** 1 Research Center for Genetic Engineering, National Research and Innovation Agency (BRIN), Bogor, 16911 Indonesia; 2 Current Address: Laboratory of Structural Virology, The International Institute of Molecular Mechanisms and Machines (IMOL), Polish Academy of Sciences, Warsaw, Poland; 3 Directorate of Laboratory Management Research Facilities, Science and Technology Park, National Research and Innovation Agency (BRIN), Bogor, 16911 West Java Indonesia

**Keywords:** HPV 52, Capsid protein L1, Yeast recombinant protein production, VLP-based vaccine, Reverse vaccinology, Expression optimization, Multi-epitope-based vaccine

## Abstract

**Background:**

Vaccination is the one of the agendas of many countries to reduce cervical cancer caused by the Human papillomavirus. Currently, VLP-based vaccine is the most potent vaccine against HPV, which could be produced by a variety of expression systems. Our study focuses on a comparison of recombinant protein expression L1 HPV52 using two common yeasts, *Pichia pastoris* and *Hansenula* *polymorpha* that have been used for vaccine production on an industrial scale. We also applied bioinformatics approach using reverse vaccinology to design alternative multi-epitope vaccines in recombinant protein and mRNA types*.*

**Results:**

Our study found that *P.* *pastoris* relatively provided higher level of L1 protein expression and production efficiency compared to *H.* *polymorpha* in a batch system. However, both hosts showed self-assembly VLP formation and stable integration during protein induction. The vaccine we have designed exhibited high immune activation and safe in computational prediction. It is also potentially suitable for production in a variety of expression systems.

**Conclusion:**

By monitoring the overall optimization parameter assessment, this study can be used as the basis reference for large-scale production of the HPV52 vaccine.

**Supplementary Information:**

The online version contains supplementary material available at 10.1186/s43141-023-00514-9.

## Background

Vaccination shows potential treatment for cancer inhibition caused by the Human papillomavirus (HPV) [1]. Vaccination using Gardasil 9 was shown to be effective to nearly 100% in preventing broad HPV-type-induced cervical, vulvar and vaginal diseases [2]. The existing strengths of local and regional communities to conduct massive production of the vaccine facilitated a relatively low-cost manufacturing process, which could cover the needs relatively faster. In addition, independency in vaccine production shows a preparedness to face the pandemic in particular for developing countries [3]. Moreover, it has been reported that HPV vaccination has become a national immunization program in more than 100 countries [4].

Recombinant protein-based vaccines are still continuously developed to fulfill the need in tackling HPV [5]. The major capsid protein L1 is the most exposed protein to the immune system that could assemble into virus-like particles (VLP), generating high immune responses [6, 7]. The main HPV vaccines that were licensed and commercially available (Gardasil and Cervarix) nowadays also utilize purified recombinant VLP-based systems [8].

HPV L1 protein could be expressed in various expression systems from prokaryotes, such as *E. coli*, as well as eukaryotes such as insects, plants, and yeast [9–11]. The yeast expression system is one of the most commonly used platforms for industrial production due to its ability to generate high protein titers [12, 13]. In addition, utilization of their post-translation modification features enhances protein solubility and folding [14].

Multi-epitope vaccine (MEV) is an alternative way to prevent and treat a pathogen infection, which has been continuously developed in a form of recombinant subunit protein or mRNA vaccines [15]. High efficacy, safe, and low-cost manufacturing are the main reasons for researchers to continue developing MEV [16]. Moreover, mRNA vaccines currently have been the breakout stars of the pandemic. Their demonstrated impressive protection has great application prospects and advantages [17, 18].

Several studies reported that L1 HPV52 (categorized as one of the high-risk HPV types) could easily be expressed in *P. pastoris* and *H. polymorpha* [19, 20]. However, the justification of which host is most likely preferable for L1 HPV52 expression is still not yet clear. Thus, this study focuses on the optimization, characterization, and comparison of codon optimized HPV52 in yeasts. Previously, L1 protein was expressed under strong methanol-inducible promoters AOX and MOX in *P. pastoris GS115* and *H. polymorpha* NCYC495, respectively [21]. Biomass, growth rate, clone copy number, VLP formation, and integration stability of *P. pastoris* GS115 and *H. polymorpha* NCYC495 were evaluated. We also described another point of view of an alternative multi-epitope vaccine (MEV). We applied reverse vaccinology to generate a recombinant fusion protein of the top listed epitopes that we identified in our previous study. They were recognized by B and T cell epitopes, which have high-level population coverage and potentially give broad-spectrum protection against other HPV types [22]. This study could give a wide perspective on tackling the carcinogenic pathogen HVP52 through optimum vaccine production, particularly for VLP, recombinant subunit protein, and mRNA-based vaccines.

## Methods

### Codon and mRNA structure analysis

The global consensus sequences of L1 HPV52 [22] were used as reference sequences for codon optimization. Codon analysis was performed using available data from Kazusa (https://www.kazusa.or.jp/codon/) [23], while Codon adaptation index (CAI) was evaluated for each host using the online available CAI evaluator (http://genomes.urv.es/CAIcal/) [24]. A 50 bp mRNA structure segment of the gene of interest starting from translation T + 1 from AOX and MOX was predicted by (https://rna.urmc.rochester.edu/RNAstructureWeb/Servers/Predict1/Predict1.html) [25]. The program could predict a minimum free energy (MFE) structure that reflected the mRNA stability.

### Construction and yeast transformation

To obtain an efficient translation in both *P. pastoris* and *H. polymorpha,* HPV52 L1 codon was optimized and cloned into pD902 that has AOX promoter (DNA 2.0, currently ATUM, Newark, CA). The L1 gene was subcloned into pHIPH4 that is regulated by promoter MOX and terminator tAMO. The pD902 was provided by ATUM, while pHIPH4 was kindly given by the University of Groningen. All construction was generated following basic molecular cloning [26].

The recombinant plasmid was transformed into *E. coli* DH5α, then isolated and linearized using *Nco*I and *Stu*I, respectively, for pD902_HPV52L1 and pHIPH4_HPV52L1. As much as 5000 ng of each linearized plasmid was introduced using electro-transformation in a 2-mm cuvette after a 1.5 kV/cm, 50 μF, and 129 Ω electric field pulse (5 ms resulting pulse length) [19]. The transformants were grown on YPD (1% yeast, 2% peptone, 2% dextrose, 2% agar) agar supplemented with zeocin to select for *P. pastoris* containing pD902_HPV52L1 and hygromycin for *H. polymorpha* containing pHIPH4_HPV52L1 at 30 ℃ and 37 ℃, respectively.

### Genome isolation and transformant validation

Transformants were screened using colony PCR and verified by sequencing analysis using specific primers (Table 1). The selected colonies were treated using Triton-X before being used as a PCR template [27]. Genomic DNA was isolated following a protocol described with slight modification [28]. Briefly, an overnight yeast culture was lysed with lysis buffer (50 mM Tris HCl pH7.2, 50 mM EDTA, 3% SDS, 1% ß-Mercaptoethanol). The homogenous lysate was extracted with an equal volume of PCI (1:1). Subsequently, sodium acetate buffer pH 5.2 and 0.6 V isopropanol were added to precipitate the DNA. The precipitated DNA was then washed with 70% ethanol and re-suspended in nuclease-free water (NFW) with 50 mg/ml RNase.

**Table 1:** Primer information that was used in this study

Primer	Utility	DNA Sequence (5′-3′)	Amplicon size (bp)
Hans_HPV52 L1_F	Gene amplification	AAGCTTATGGTGCAAATTCTGTTCTATATTCTCGTGATC	1602
Hans_HPV52 L1_R		GTCGACTTAACGTTTTACCTTTTTTTTTTTGGTCGAAGTTCT	
pMOX_F	Integrant analysis	ACGTGACCTTGCCTAACCG	2062
tAMO_R		TTATTTACCGCAACAAGAGC	
qPCR_HPV52 L1_F	Integration Stability Test	GTATTTCAGGACACCCACTGC	83
qPCR_HPV52 L1_R		GTTGTCGATACCTGGCTTACC	
ARG4_F	Copy number analysis	TCCTCCGGTGGCAGTTCTT	84
ARG4_R		TCCATTGACTCCCGTTTTGAG	
ACT1F	Copy number analysis	TCCAGGCTGTGCTGTCGTTG	139
ACT1R		CCGGCCAAGTCGATTCTCAA	

### Protein expression, isolation, SDS PAGE, western blot (WB), and transmission electron microscopy (TEM)

The best performing colony was chosen by the expression level of each clone. The expression was performed in a shake flask system with 1:10 aeration. Yeast was grown in Buffered Glycerol Complex Medium (BMGY) to produce a high biomass yield and then transferred into Buffered Methanol-Complex Medium (BMMY) with methanol added as an inducer at the optimal concentration for each strain: 0.5% (*P. pastoris*), and 1% (*H. polymorpha*). Growth kinetics analysis was performed by measuring OD_600_ values every 24 h until 96 h at 22 ℃ and 30 ℃ for *P. pastoris* cultures, while *H. polymorpha* cultures were measured at 30 ℃ and 37 ℃ [29]. The analysis was performed in three biological replicates.

Protein was isolated using a glass bead added to lysis buffer (50 mM sodium phosphate, pH 7.4, 1 mM phenylmethylsulfonyl fluoride (PMSF), 1 mM EDTA, and 5% glycerol). The sample was separated using 12% SDS–PAGE under reducing condition, 400 mA for 90 min. The gel was transferred into the nitrocellulose membrane using the wet transfer method. The membrane was blocked using skim milk for 1 h. Next, the primary polyclonal antibody L1 HPV52 (Creative Diagnostic, CABT B8799) and anti-rabbit conjugated HRP (secondary antibody) were subsequently added at 1:20 polyclonal antibody L1 HPV52 (Creative Diagnostic, CABT B8799). Finally, the specific protein band was visualized by pouring TMB chromogenic substrate. The isolated protein was also evaluated by immunoblot.

For TEM analysis, the first step protein purification was conducted by 50% ammonium sulfate precipitation, followed by overnight dialysis. The samples were absorbed on carbon-coated copper grids and negatively stained with 2% phosphotungstic acid. The grids were air-dried before examination under a transmission electron microscope, JEOL 1010, 80 kV.

### Copy number analysis and protein quantification

Copy number of integrated HPV52 L1 gene in both yeasts was determined using qPCR MyGo Pro RT-PCR with three-step amplification for 40 cycles of initiation (98 ℃, 2 min), denaturation (98 ℃ 10 s), annealing (60 ℃, 10 s), and extension (68 ℃, 30 s). Additional melting curve analysis was added in the final step. Transcription of reporter gene ARG4 and ACT in *P. pastoris* and *H. polymorpha* were respectively detected using specific primers as described in Table 1 [30, 31]. Comparison of copy number was normalized by the lowest CT value. In addition, PCR efficiency was calculated by the equation below [32]. The analysis was performed using three biological replicates.

$$\%\,\mathrm E\mathrm f\mathrm f\mathrm i\mathrm c\mathrm i\mathrm e\mathrm n\mathrm c\mathrm y=\left(10^{\left(-\frac1{\mathrm{slope}}\right)}-1\right)\;\times\;100$$

The total amount of lysate and precipitated fraction (ammonium precipitation) were quantified by BCA Protein Assay Kit (Thermo Scientific™). The HPV52 L1 dot blot was detected using polyclonal antibody L1 HPV52 (Creative Diagnostic, CABT B8799). The concentration of L1 protein was quantified using a densitometer, compering with recombinant purified HPV52 L1 in different concentrations as a standard. ELISA was also performed to specify the amount of HPV52 L1 using a monoclonal antibody (Creative diagnostics, CABT-B8810).

### Epitope-based vaccine design and validation

The top highest antigenicity level of listed epitopes was selected based on our previous study (Supplementary Table S1). The designed vaccine was connected using EAAAK that separated the entire epitope and the other supporting components. Meanwhile, AAY and GPGPG linkers were used to connect individual B and T cell epitopes, respectively. The 50S ribosomal protein L7/L12 (Locus RL7_MYCTU) derived from *Mycobacterium tuberculosis* was also added to boost the vaccine immunogenicity. Antigenicity of the whole construct, immune response, toxicity, antigenicity, and physicochemical were evaluated using Vaxijen [33], C-ImmSim [34, 35], Toxinpred [36], and Protparam [37], respectively.

### Designed mRNA vaccine docking and immune simulation

Docking analysis was performed using the available online server The ClusPro 2.0 server [38]. The protein structure of the designed vaccine (Receptor) was predicted using I-TASSER [39] and then validated by Ramachandran (ZLab server (https://zlab.umassmed.edu/bu/rama/index.pl). The TLR4 (PDB ID: 4G8A) was applied as protein ligan, and its interaction with the receptor was identified, using pdbsum [40]. All protein structures are visualized by pymole software.

### Statistical analysis

Statistical analysis was performed using GraphPad Prism (GraphPad Software, San Diego, CA) by applying Student’s *t* test to determine differences between the two-group data. The data with *p* < 0.05 were considered to have significant differences.

## Result

### Gene design, synthesis, and vector expression construction

**Fig. 1:**
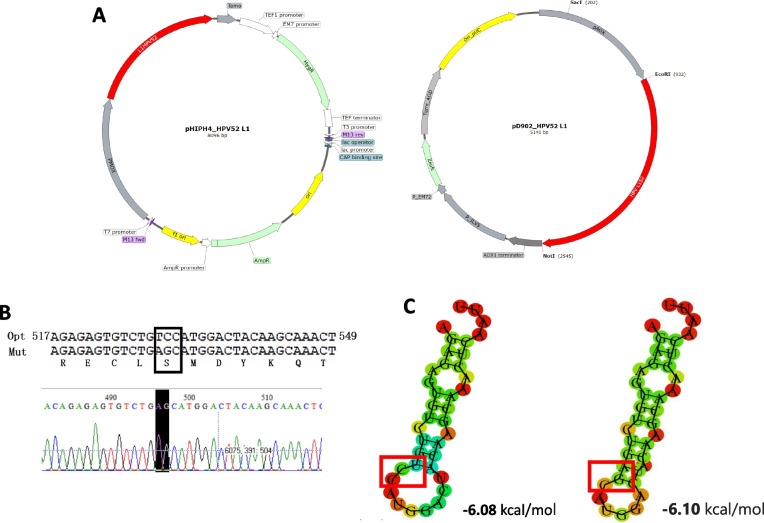
Vector construction, synonymous mutation, and mRNA structure analysis. **A** Construction of gene expression vectors. PHIPH4_HPVL152 for *H. polymorpha* (left) and pD902_HPVL152 for *P. pastoris* (right). **B** Synonymous mutation of serine residue was found in HPV52 L1 isolated sequences from all *H. polymorpha*. **C** mRNA structure around the mutation site of HPV52 L1 was not significantly different

The optimized codon was shown to be feasible for both *P. pastoris* and *H. polymorpha* with an adaptation coefficient of 0.77. The RNA structure of the optimized codon could provide a higher stability by decreasing folding free for more preferable transcription (Supplementary Figure S1) [41]. The gene encoding L1 HPV52 was inserted into the multi-cloning site between *BamH*I and *Not*I for pD902 and *Hind*III and *Sal*I for pHIPH4 (Fig. 1A). Synonymous mutation of serine (TCC to AGC) was found in all positive clones from *H. polymorpha* (Fig. 1B), with slightly different mRNA free energy (Fig. 1C). Meanwhile, *P. pastoris* showed matched sequences with the designed codon in all positive colonies. The synonymous mutation found in clone *H. polymorpha* did not affect the reading frame of the HPVL152 protein. In addition, the shape of mRNA is relatively similar which will not interfere with the normal interaction with the ribosome which allows the production of similar protein levels.

### Growth kinetics and expression level of HPV52 L1

**Fig. 2:**
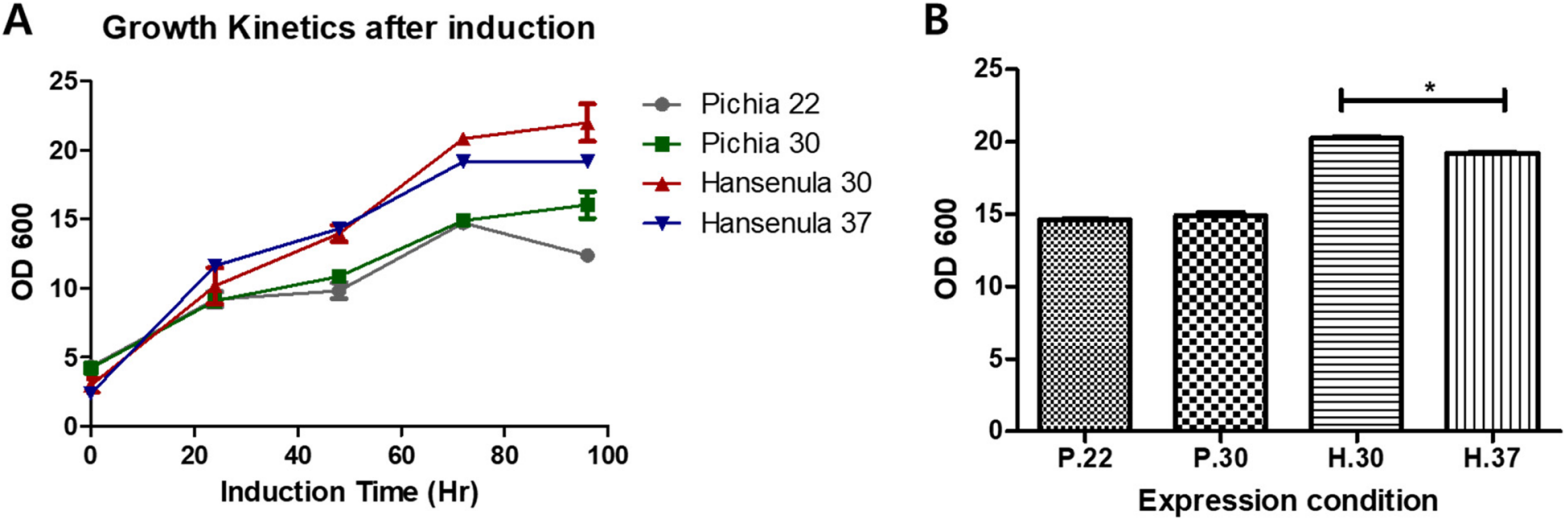
Biomass analysis of HPV52 L1 expression. **A** Growth curve after induction. **B** Differences of final OD between two hosts. *n* = 3, *p* < 0.05 were considered to have significant differences

**Fig. 3:**
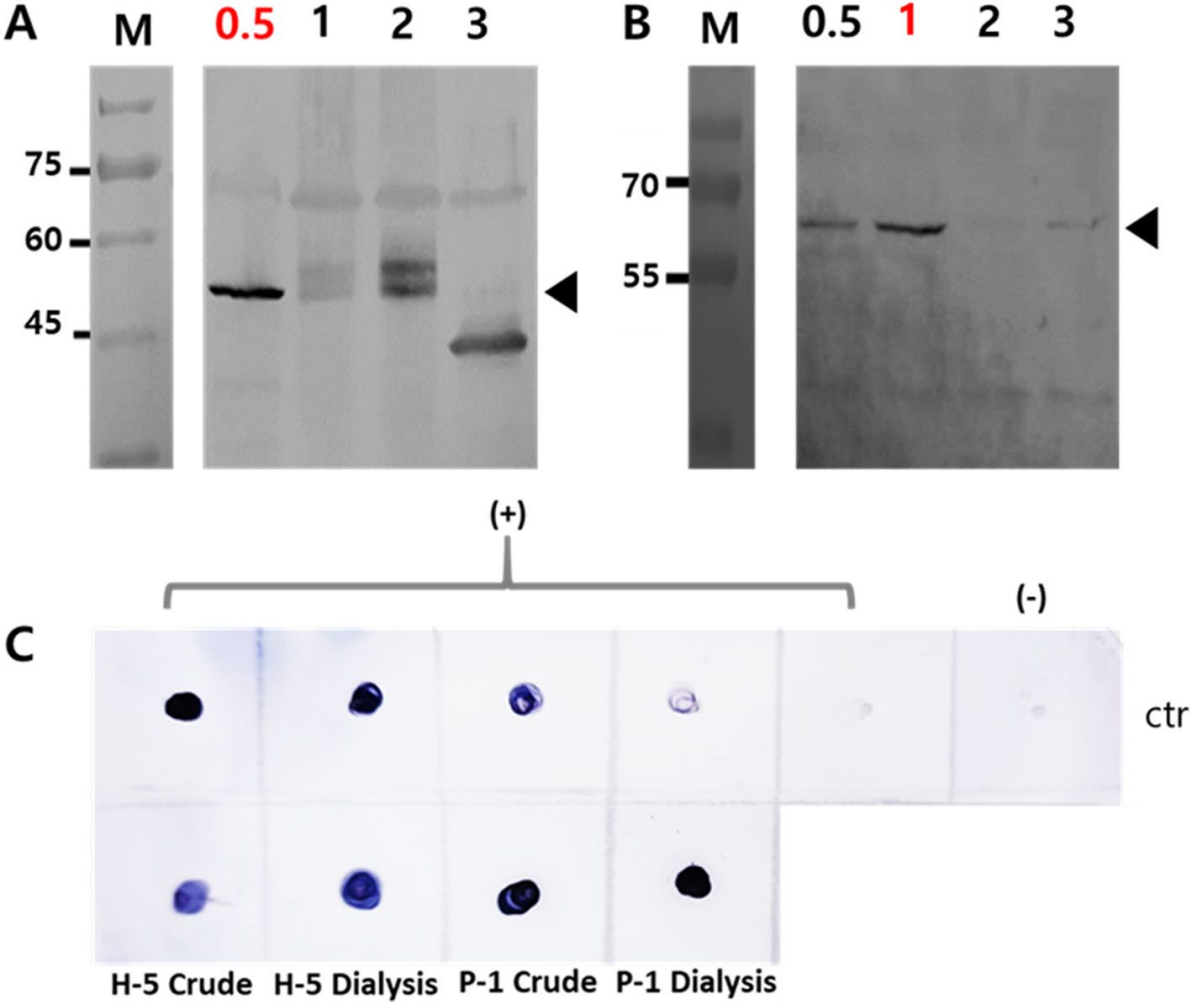
Expression profile of HPV52 L1 induced by different amounts of methanol.** A** WB profile of HPV52 L1 expression in *P. pastoris.* **B** WB profile of HPV52 L1 expression in *H. polymorpha.* **C** Immunoblot of HPV52 L1 expression using polyclonal antibody. H-5 indicated *H. polymorpha* colony 5, while P-1 represents *P. pastoris* colony 1, the protein was isolated from 72 h culture in the optimum condition

*H. polymorpha* showed a significantly different growth rate between two temperature conditions while *P. pastoris* did not (Fig. 2A). It correlates with the biomass production from *H. polymorpha* that showed significantly distinction between the two conditions while *P. pastoris* was observed with no change (Fig. 2B). However, they both reached a stationary phase after 72 h with the highest biomass levels observed at 30 ℃ (Fig. 2A).The optimum expression was observed at 30 ℃ with 0.5% inducer for *P. pastoris* and 37 ℃ with 1% inducer for *H. polymorpha* (Fig. 3A, B)*.*

WB and immunoblot analyses showed the expression level of ~ 59 kDa HPV52 L1 in *P. pastoris* was ~ 1.5 times higher than *H. polymorpha* (detected by ELISA) (Fig. 3C). In addition, the percentage protein recovery of *P. pastoris* also exhibited ~ 1.4 times higher than *H. polymorpha* even though the protein recovery from ammonium precipitation were similar in both yeasts (Table 2).

**Table 2:** Comparison of concentration L1 HPV 52 and protein recovery

Step	Total protein (mg)		ELISA (ug)	
	Hp	Pp	Hp	Pp
**Lysate supernatants**	76.49	73.83	630	840
**(NH)**_**2**_**SO**_**4**_ **precipitation**	14.62	14.23		
**Recovery (%)**	19.11	19.27	4.31	5.90

Total protein was determined by BCA protein assay. *Hp H. polymorpha*, *Pp P. pastoris*. % protein recovery was determined by comparing ELISA and (NH)_2_ SO_4_ precipitation values

### Copy number and stability during methanol induction

**Fig. 4:**
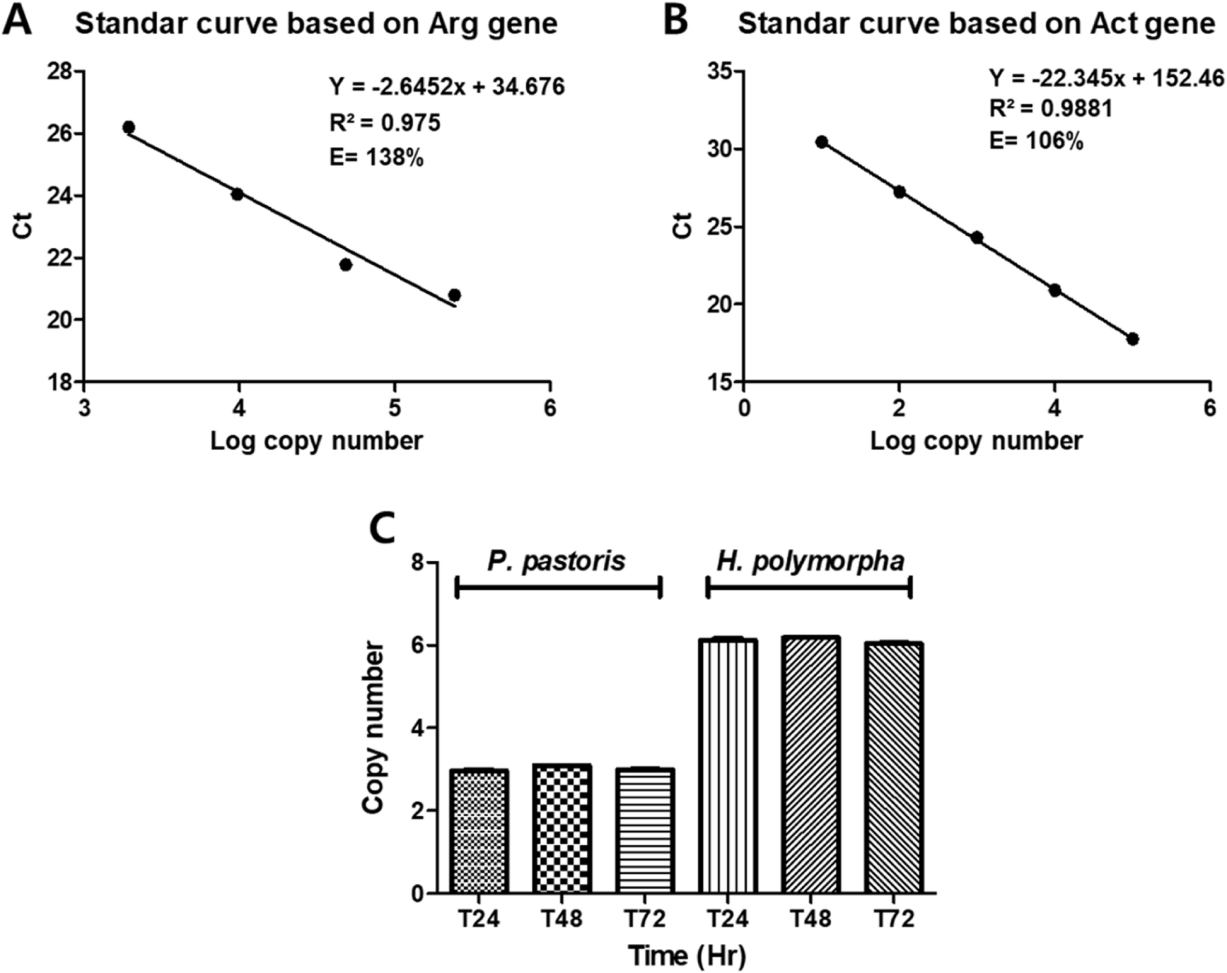
Standard curve and stability analysis. **A** Standard curve of the reporter gene, ARG4 in *P. pastoris*. **B** Standard curve of the reporter gene, ACT in *H. polymorpha*. **C** Stability of HPV52 L1 copy number in each host during methanol induction. The protein expression was induced using an optimal amount of inducer

The isolated genome of each clone was serially diluted and set as a template for copy number analysis. The *r*^2^ regression value of ACT and ARG showed a good correlation with *r*^2^ > 0.9 (Fig. 4A, B). The melting curve also showed specific amplification, described by a single peak from all samples (data not shown). The best performing colony stably expressed the protein during methanol induction (Fig. 4C). The fact that both hosts have a stable copy number during induction indicated a stable integration leading to a stable expression [42].

### TEM analysis

**Fig. 5:**
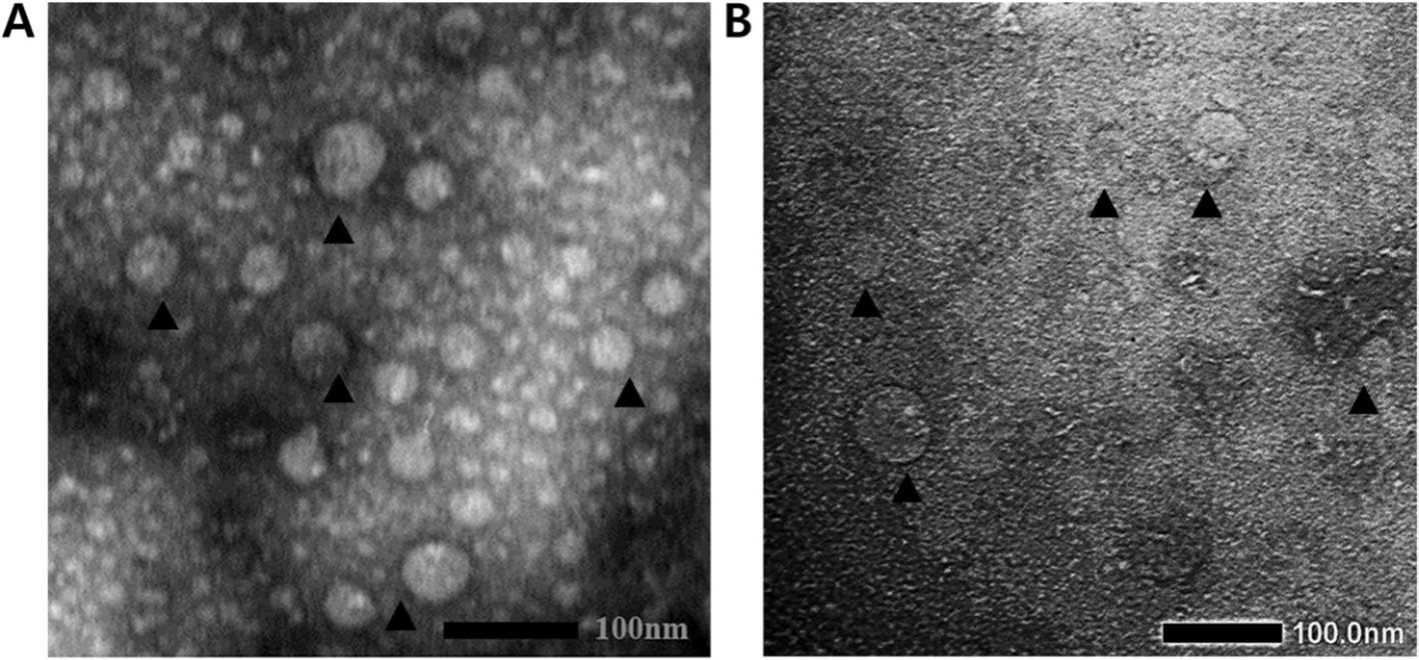
TEM images showing self-assembled VLP of HPV52 L1. **A** VLP formation of HPV52 L1 was obtained from *P. pastoris* dialyzed crude. **B** VLP formation of HPV52 L1 was obtained from *H. polymorpha* dialyzed crude. View examples of VLP are indicated by arrows

The benefit of using a yeast expression system is the ability to form self-assembly VLP. VLP formation was observed by TEM with approximately 50 nm size-like nature virions (Fig. 5). The unsynchronized form was commonly found at the protein expression without further purification, which can be caused by differential expression and VLP assembly periods. To increase homogeneity and stability of VLP disassembly and reassembly VLP is still required.

### Vaccine design and validation

**Fig. 6:**
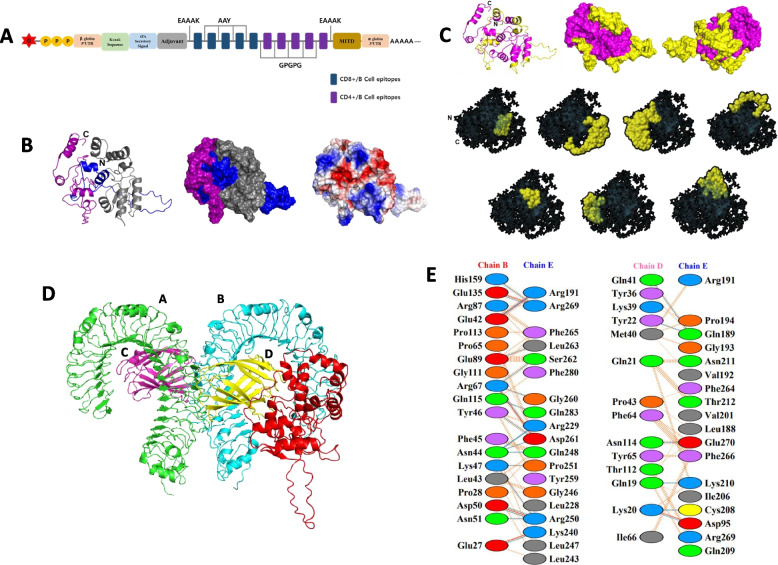
The designed vaccine and epitope mapping and interaction. **A** Scheme of mRNA vaccine design. **B** Surface and charge structure of multi-epitope recombinant vaccine HPV 52, the color is according to the vaccine scheme. **C** Linear (upper, indicated in yellow) and conformational B (lower, indicated in yellow) cells epitope mapping showed the overlapping region with T cells epitope. **D** Docking analysis of TLR4 and recombinant vaccine. **E** Interaction mapping residues of TLR4 and a recombinant vaccine

The predicted protein structure of vaccine design showed 85% Highly Preferred Conformations (Supplementary Figure S2). It also showed a stable expression, high solubility, and nontoxic features (Supplementary Table S2). The probable antigenicity of the designed vaccine exhibited slightly higher than the capsid protein itself in VaxiJen simulation (0.50 > 0.48). It covered B and T cell epitopes (Fig. 6A), with charge distribution profile (Fig. 6B) and B cell surface recognition site distributed across the whole structure (Fig. 6C). It was reported that this epitope potentially gives a cross-protection profile across other HPV types. Moreover, the vaccine is also considered can cover a wide region of the population, nontoxic, stable, and non-allergenic as well [22].

### Docking analysis designed mRNA vaccine to TLR

No less than 29 complexes formation was generated by Claspro with the lowest energy of – 1283 selected as the best complex (Fig. 6D). The complex formation between the designed vaccine and TLR4 is stabilized by 1 salt bridge and 6 hydrogen bonds in chain D of TLR4 and 5 salt bridges and 20 hydrogen bonds in chain B (Fig. 6E). Their interaction is facilitated by the net charge of the contacting area between the ligand-receptor.

### Immune simulation

**Fig. 7:**
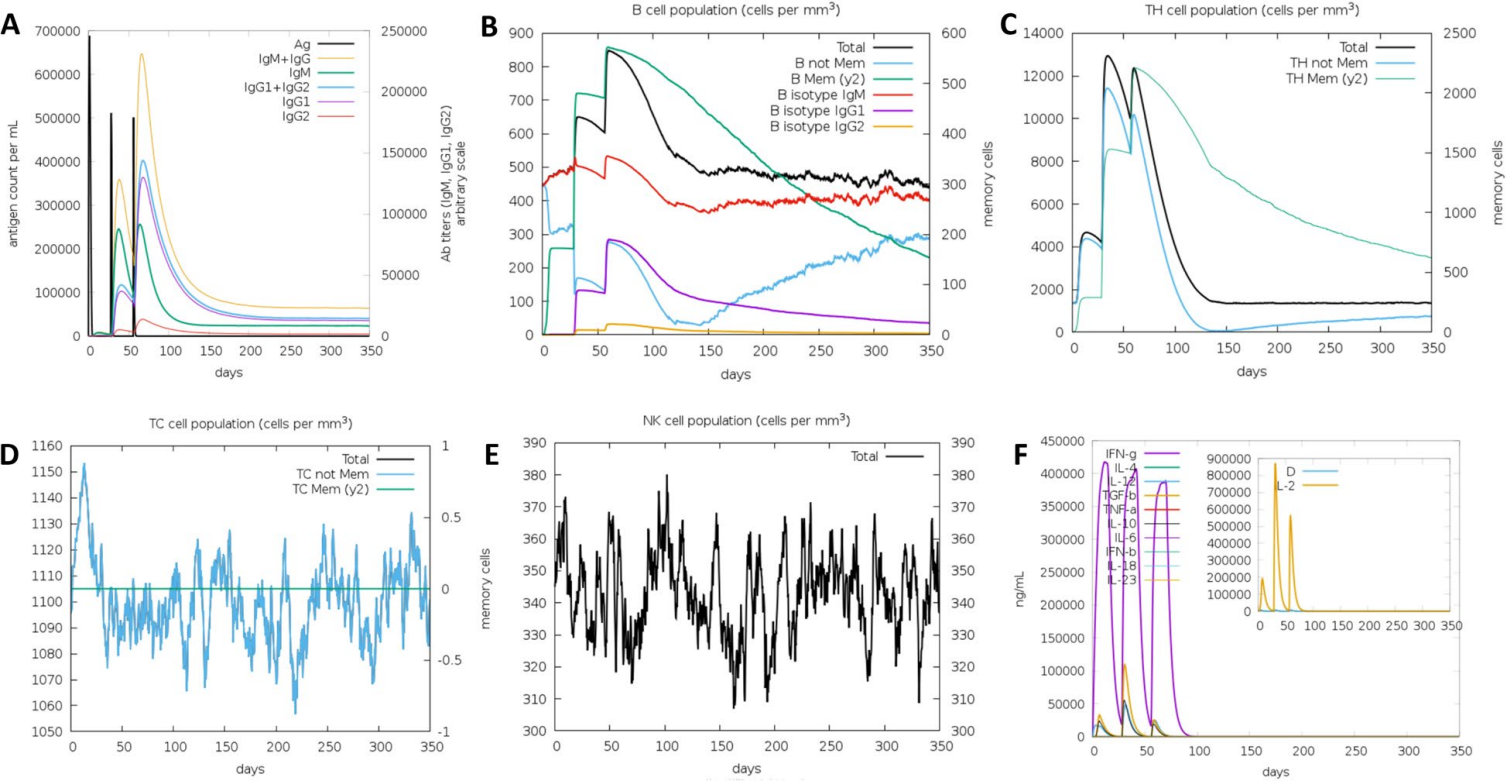
Vaccine immune simulation using C-ImmSim. **A** Immunoglobulin production in response to antigen exposures, **B** B cell population, **C** T helper cell population, **D** Total production of T-cytotoxic cells, **E** natural killer cells production, and **F** cytokine level profile after the injections

Immune response was increased following antigen exposures, it showed that secondary and tertiary responses were higher than primary induction (Fig. 7A). Immunoglobulin response of IgM showed a higher level than IgG in the primary induction. In the second and third doses, the IgG1 + IgG2, as well as IgG1, exhibited a higher level than IgM along with antigen reduction. The increasing level of B, Th (helper), Tc (cytotoxic), and NK (Natural Killer) cells were also observed for a long period which indicated memory formation (Fig. 7B–E). In addition, the IFN gamma showed robust activation during vaccination with a variety of immune responses indicated by a lower Simpson index (Fig. 7F). The data suggested that the designed vaccine has full filled good vaccine indicators and can be considered for further in vitro and in vivo analysis.

## Discussion

Recombinant protein expression has been used extensively to produce vaccines for a long time. This also applies to the vaccine manufacture against HPV which mostly use major capsid protein L1. It is the most exposed protein that can stimulate immune response similar to the native virion [7]. Yeast is the one of the best platforms for producing viral-like particle for HPV. In addition to the self-assembly HPV VLPs, the ability of yeasts to produce large amounts of protein has always been advantage for the industry [43].

In this study, we highlighted two different approaches to produce HPV vaccine. First, we optimized recombinant expression L1 HPV52 using two different yeast (*P. pastoris* and *H. polymorpha*) and took advantage from their self-assembly VLP system. We also compared their profile in the optimized condition which include growth kinetics, biomass, temperature, inducer amount, copy number stability, and VLP formation. The second approach is to use the potential antigenic peptide from our study to generate peptide-based vaccine in the form of recombinant or mRNA vaccines.

Selecting the right host strain is the first step in opening the bottleneck for protein production. We used yeast expression system to produce heterologous protein that have been used for industrial and biopharmaceutical in the large amount for many years. Yeast is easy to be manipulated genetically, short generation times, large scalable using fermentation, less expensive, and suitable for various proteins that needs post translation modification [44].

We optimized codon preference for yeast to enhance the protein production. By replacing rare codons to match with natural host codons leads to proper protein folding by preserving slow translation regions [45]. We also used AOX and MOX which are categorized as strong methanol inducible promoters to give a higher level of heterologous protein expression [45].

Increasing cell biomass as a strategy to elevate protein yield has been extensively studied by manipulating the host and its environments such as medium or feeding strategy [46, 47]. Our research using a shake flask system showed that *H. polymorpha* gave a higher biomass compared to *P. pastoris*. Based on our data, biomass has not significant effect on the L1 HPV52 expression. It was also suggested that accumulation of biomass does not necessarily linear with protein production [47]. Because our study used shake flask system which has limitations in controlling methanol uptake, we used the same aeration and methanol concentration throughout the production. It decreased methanol and oxygen shortage that required by higher cell [48]. This may result differently once the feed-batch strategy is implemented at the fermenter scale.

High copy number integrant in yeast could enhance protein titers. However, as with biomass, copy number does not always have a directly proportional correlation to the protein titer. An inverse correlation was demonstrated in our study by *P. pastoris* having higher expression level of L1 HPV52 in a relatively low copy number. This is in line with the number of inducers indicating that the lowest inducer gave a higher expression level. It might be that *P. pastoris* requires a slow production of L1HPV52 to reduce metabolic stress. In contrast for *H. polymorpha* was relatively more resistance to foreign gene (in this context L1 HPV52), causing its expression to be higher at a relatively higher copy number. However, both yeasts showed stabile integration during protein production.

The overexpression of heterologous protein may cause stress on a secretory pathway, enhancing the unfolding protein response that leads to protein degradation and other cellular stress responses [49]. Moreover, the combination of transcription and translation levels, as well as protein folding play a role in the protein features like solubility and stability that affect the protein function [50, 51].

VLP formation correlates to protein folding and its environmental properties to minimize free energy in higher structures [52]. Nevertheless, this study showed reproducible data with previous reports that confirmed the self-assembly of the VLPs. For medical application purposes, further purification steps and subsequent VLPs reassembly are required to obtain the correct particle size at the appropriate amount of yield to induce an adequate immune response [3, 53–55].

Recombinant antigen protein purification for biopharmaceutical product should not have any additional component that may affect the biological system (safety issue). We used non-tagging protein purification strategy which not required additional tagging cleavage step that can increase purification efficiency. It can use size exclusion chromatography tandem with ion exchange or hydrophobic interaction chromatography. In addition, we did ammonium precipitation as initial step before further purifications. Immune response validation should also be tested in animal models, this may help in assessing which host can produce better L1 HPV52 folding as well as VLP maturation.

Until now the best, licensed HPV vaccine with high efficacy uses a VLP-based platform. It utilizes capsid protein L1 as the main component, inhibiting viral replication using the neutralizing mechanism. VLP gives a high titer of antibody production because it mimics a viral nature form that leads to undistinguished by the immune system [56].

Peptide based vaccine via recombinant protein or mRNA platform can be used as another option to produce potential vaccine. Moreover currently mRNA vaccine was become new star in infectious diseases prevention such as in tackling COVID19. We used computational approach using in silico protein modelling from our potential peptide we tested in previous study to get initial insight for the real biological event.

In general vaccine could be recognized by pathogen recognition receptors (PRRs), mainly expressed by cells of the innate immune system. VLP internalization prompts cell maturation and epitope presentation through major histocompatibility complex (MHC) class I or class II molecules [57]. MHC I bind to CD8^+^ T cells responsible for the cytotoxicity activity, while MCH II binds to CD4^+^ T cells bridging antibodies production by B cells.

The mRNA normally produced by in vitro transcription and then delivered by vector-mediated internalization using a lipid, nanoparticle, or polymer-based delivery system into the body [58]. mRNA is translated by cellular machinery in the cytoplasm and then undergoes posttranslational modification to stabilize the tertiary structure to have fully functional protein properties. MHC I-targeting domain (MITD) which is present in the construct responsible for peptide secretion through transporting the peptide to particular compartments in the endoplasmic reticulum and Golgi body, then MHC-I and MHC-II could present them on the surface of cells [59].

**Fig. 8:**
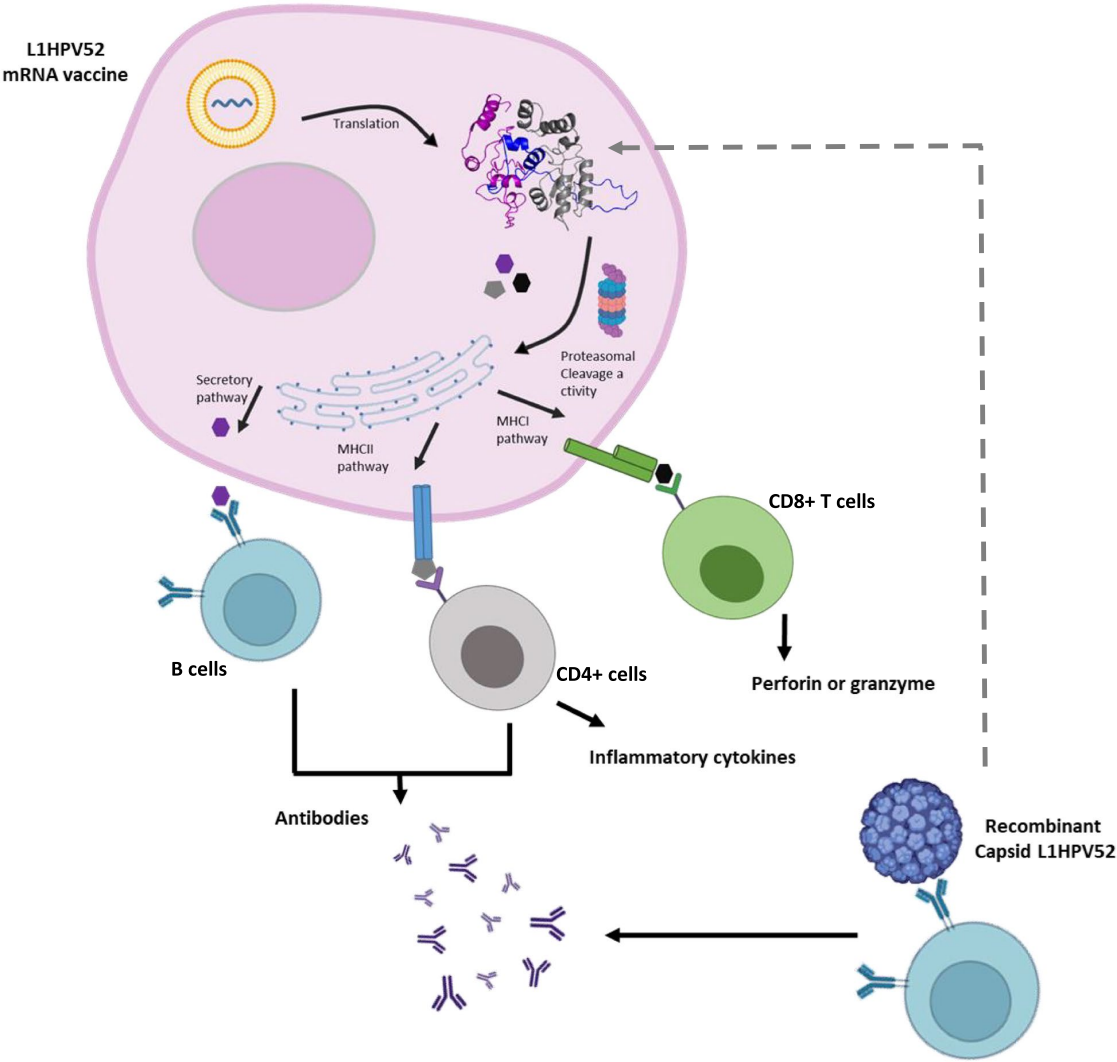
Purposed mechanism of action of VLP, mRNA, and multi epitope protein-based vaccines. The schema was created using Biorender (https://biorender.com/)

The right linker should be added to avoid inter-domain interaction leading to impaired bioactivity [60]. A balance between flexibility and rigidity that maintain a stable conformation when expressed is required [61]. mRNA ORF required an extra region for the polymerase to stay and start the transcription. We used a common *Xenopus* beta globulin in 5′ and alpha 3′ UTR that flank the mRNA ORF which was reported to significantly enhance the stability of mRNA as well as protein translation [62]. The open reading frame from the first epitope to the end with linker in between can be used for recombinant subunit vaccine using common protein expression. In addition, adjuvant is also a key point that could enhance the immune response by several mechanisms such as depot formation, recruitment of immune cells, induction of cytokines and chemokines, enhancement of antigen uptake, presentation, and transport to draining lymph nodes [63]. All the vaccine mechanism of action is summarized in Fig. 8.

In general, our construct was computationally predicted have a good vaccine properties. It has a good solubility index and potentially induce immune response, however in vitro and in vivo validation is still needed.

## Conclusion

Our study showed that the expression system of *P. pastoris* GS115 and *H. polymorpha* NCYC495 allowed the self-assembly of VLPs into a correct human papilloma pseudovirion structure. *P. pastoris* tend to give a higher level of L1 protein than *H. polymorpha* in a batch system; however, both hosts gave a stable integration that keeps the protein expression stable during production. Our purposed vaccine predicted has a high immune activation, is safe and is easy to produce in various expression systems. However, the designed vaccine needs to validate further in vitro and in vivo assay. In the end, a combination of different parameters in characterizing protein expression is a powerful way of exploring protein expression profiles that could be helpful for massive production industrial-scale production.

## Supplementary Information

**Additional file 1: Table S1. **Listed epitopes included in the construct. **Figure S1.** mRNA structure of HPV52 L1 starting from predicted transcription initiation complex. A mRNA structure of the native codon P. pastoris. B mRNA structure of the optimized codon P. pastoris. C mRNA structure of the native codon H. polymorpha, D mRNA structure of the optimized codon H. polymorpha. **Figure S2.** The Ramachandran plot of predicted vaccine structure using ZLab server; green: highly preferred conformations, delta ≥ −2; brown: preferred conformations, −2 > delta ≥ −4; and red: questionable conformations, delta < −4.

## Data Availability

All data generated or analyzed during this activity are included in this published article.

## Abbreviations

HPV: Human papilloma virus
VLP: Virus-like particles
MEV: Multi epitope vaccine
CAI: Codon adaptation index
MFE: Minimum free energy
PCR: Polymerase chain reaction
NFW: Nuclease-free water
SDS-PAGE: Sodium dodecyl sulfate polyacrylamide gel electrophoresis
WB: Western blot
TEM: Transmission electron microscope
BMGY: Buffered glycerol complex medium
BMMY: Buffered methanol-complex medium
PMSF: Phenylmethylsulfonyl fluoride
BCA: Bicinchoninic acid
EDTA: Ethylenediaminetetraacetic acid
TMB: 3,3′,5,5′-Tetramethylbenzidine
PRRs: Pathogen recognition receptors
MHC: Major histocompatibility complex
MITD: MHC I-targeting domain
TLR: Toll-like receptor
