# Supplementary material for: Optimization, characterization, comparison of self-assembly VLP of capsid protein L1 in yeast and reverse vaccinology design against human papillomavirus type 52
Source: J Genet Eng Biotechnol. 2023 May 24;21:68. doi: 10.1186/s43141-023-00514-9 (PMC10206359; doi:10.1186/s43141-023-00514-9)
Supplement: Supplementary file 1 — Additional file 1: Table S1. Listed epitopes included in the construct. Figure S1. mRNA structure of HPV52 L1 starting from predicted transcription initiation complex. A mRNA structure of the native codon P. pastoris. B mRNA structure of the optimized codon P. pastoris. C mRNA structure of the native codon H. polymorpha, D mRNA structure of the optimized codon H. polymorpha. Figure S2. The Ramachandran plot of predicted vaccine structure using ZLab server; green: highly preferred conformations, delta ≥ −2; brown: preferred conformations, −2 > delta ≥ −4; and red: questionable conformations, delta < −4. [file 43141_2023_514_MOESM1_ESM.docx]

**SUPPLEMENTARY FIGURE LEGENDS**

Table S1 Listed epitopes included in the construct.

| No | Sequences |
| --- | --- |
| MHC I epitopes | |
| 1 | LPDPNKFGF |
| 2 | YVSRTSIYY |
| 3 | FVTVVDTTR |
| 4 | ATILEDWQF |
| 5 | KVSGLQYRV |
| MHC II epitopes | |
| 1 | YRFVTSTAITCQKNT |
| 2 | EPYGDSLFFFLRREQ |
| 3 | GRKFLLQAGLQARPK |
| 4 | PYGDSLFFFLRREQM |
| 5 | RKFLLQAGLQARPKL |

Table S1 Listed epitopes included in the construct.

| Formula | C_1420_H_2221_N_367_O_411_S_4_ |
| --- | --- |
| Theoretical pI | 7.66 |
| Molecular weight | 31138.71 |
| Aliphatic index* | 81.68 |
| Grand average of hydropathicity (GRAVY)** | -0.098 |
| The instability index (II) | 29.23 |
| half-life | 30 hours (mammalian reticulocytes, in vitro) |
|  | >20 hours (yeast, in vivo). |
|  | >10 hours (Escherichia coli, in vivo). |
| Toxicity | Nontoxic |

*a stable protein is indicated by an instability value > 40

**positive GRAVY index indicates hydrophobic protein while negative indicates it is hydrophilic.


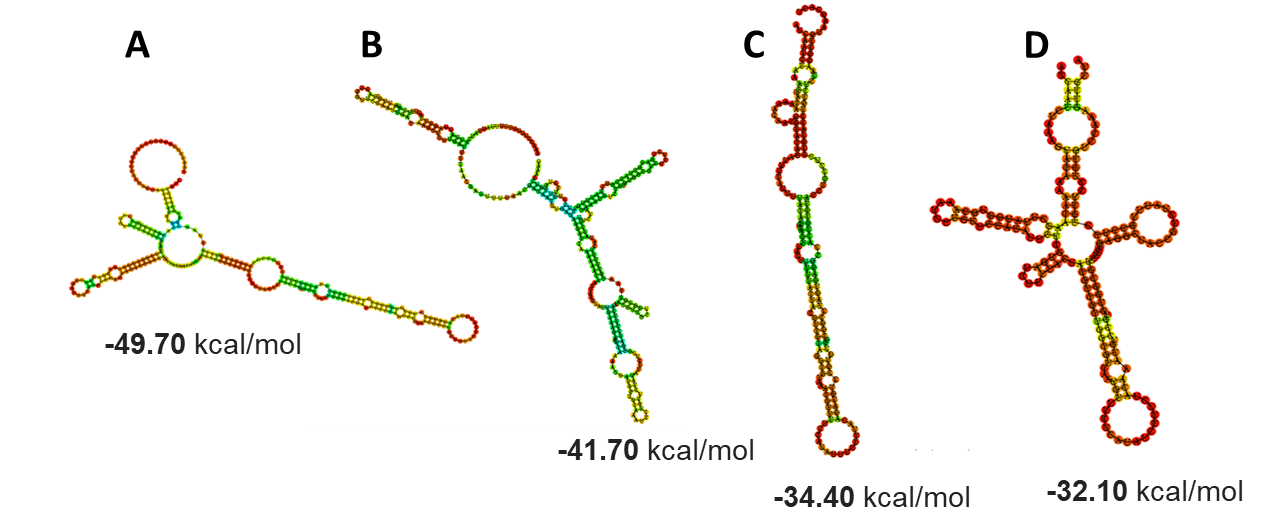


**Fig. S1**. mRNA structure of HPV52 L1 starting from predicted transcription initiation complex. **A** mRNA structure of the native codon *P. pastoris.* **B** mRNA structure of the optimized codon *P. pastoris.* **C** mRNA structure of the native codon *H. polymorpha*, **D** mRNA structure of the optimized codon *H. polymorpha*.


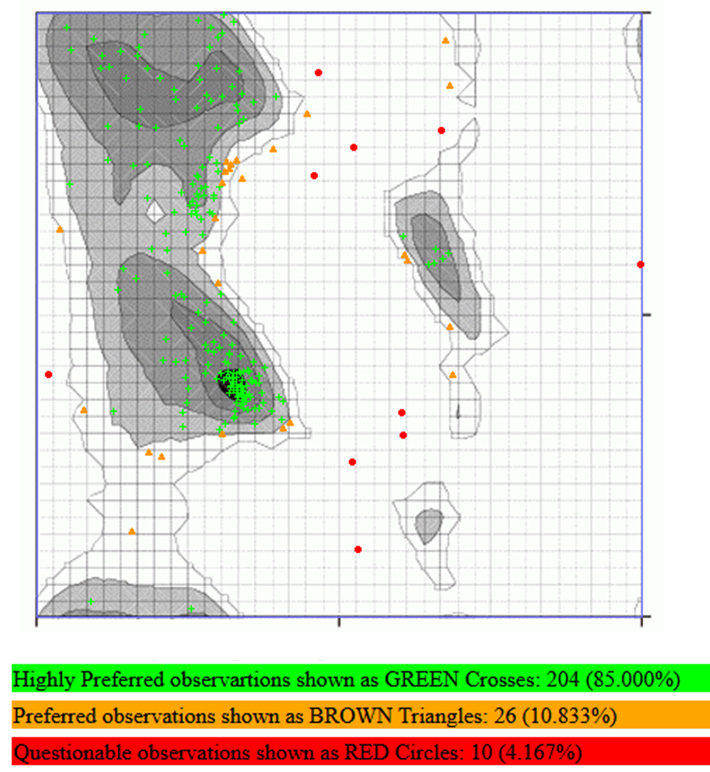


**Fig. S2**. The Ramachandran plot of predicted vaccine structure using ZLab server; green: highly preferred conformations, delta ≥ −2; brown: preferred conformations, −2 > delta ≥ −4; and red: questionable conformations, delta < −4.
